# 

**Published:** 1894-05-19

**Authors:** 


WITH EXTRA NURSING SUPPLEMENT.
Per Annum, 8s. 6d., post free.
Monthly Parts, 6d.; post free, 8d.
Ditto per Annum, 8s., post free.

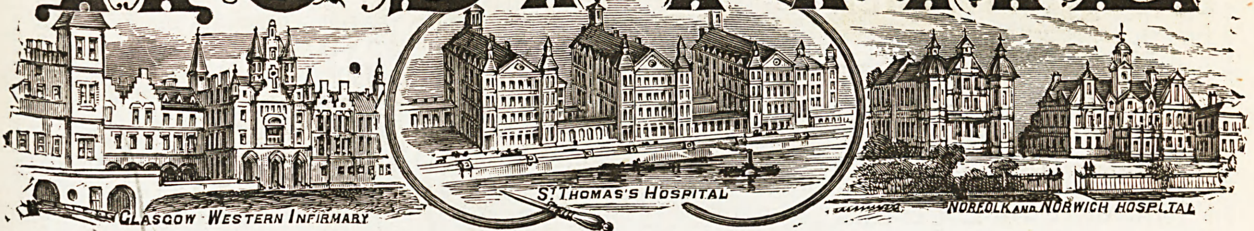

No. 399. Vol. XVI. WITH EXTRA NURSING SUPPLEMENT. SATURDAY, MAY 19TH, 1894.

